# *Eclipta prostrata* (L.) L. (Asteraceae): Ethnomedicinal Uses, Chemical Constituents, and Biological Activities

**DOI:** 10.3390/biom11111738

**Published:** 2021-11-22

**Authors:** Deepak Timalsina, Hari Prasad Devkota

**Affiliations:** 1 Central Department of Chemistry, Tribhuvan University, Kathmandu 44618, Nepal; geniusdipu5@gmail.com; 2 Graduate School of Pharmaceutical Sciences, Kumamoto University, 5-1 Oe-honmachi, Chuo-Ku, Kumamoto 862-0973, Japan; 3 Headquarters for Admissions and Education, Kumamoto University, 2-40-1 Kurokami, Chuo-Ku, Kumamoto 860-8555, Japan

**Keywords:** *Eclipta prostrata*, *Eclipta alba*, ecalbasaponin, hepatoprotective, wedelolactone

## Abstract

*Eclipta prostrata* (L.) L. (Syn.: *Eclipta alba* (L.) Hassak, Family: Asteraceae) is an important medicinal plant in the tropical and subtropical regions. It is widely used in treating various diseases of skin, liver and stomach in India, Nepal, Bangladesh, and other countries. The main aim of this review was to collect and analyze the available information on traditional uses, phytoconstituents, and biological activities of *E. prostrata*. The scientific information was collected from the online bibliographic databases such as Scopus, MEDLINE/PubMed, Google Scholar, SciFinder, etc. and books and proceedings. The active phytochemicals were coumestan derivatives, phenolic acid derivatives, flavonoids, triterpenoid and steroid saponins, substituted thiophenes, etc. Various extracts and isolated compounds of *E. prostrata* showed a wide range of biological activities such as antimicrobial, anticancer, hepatoprotective, neuroprotective and hair growth promoting activities. Relatively a few studies have been performed to reveal the exact phytoconstituents responsible for their corresponding pharmacological activities. Future studies should focus on detailed mechanism based studies using animal models and clinical studies.

## 1. Introduction

The use of plants in traditional medicines covers a wide range of therapeutic uses to treat the infection as well as many chronic diseases [1,2,3,4]. Many people still rely on the traditional medicine and healthcare because of their wider cultural acceptance and affordability [5]. The plant based bioactive compounds have been an important source of modern drugs discovery and development [6]. Hence, the medicinal value of various plants should be explored with their pharmacological significance and potential application in different products.

**Figure 1:**
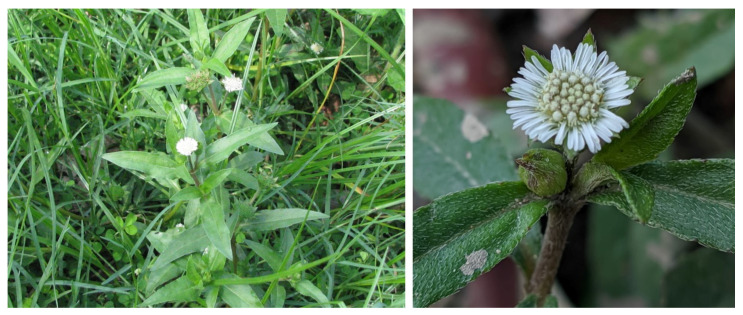
Photographs of *Eclipta prostrata* (Photos by Basu Dev Neupane, used with permission).

**Figure 2:**
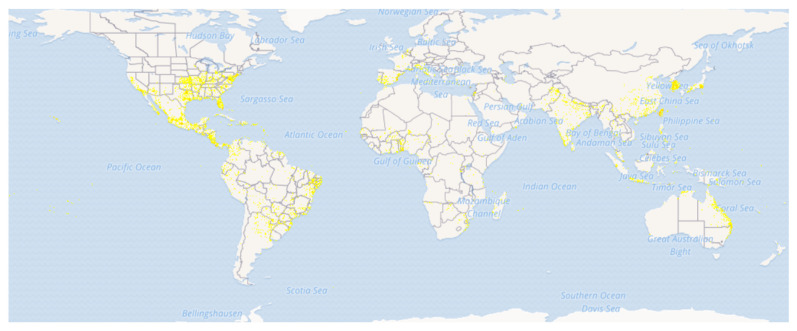
Distribution map of *Eclipta prostrata.* (Source: GBIF, https://www.gbif.org/species/5384950 (accessed on 1 November 2021) [15]).

*Eclipta prostrata* (L.) L. (Syn.: *Eclipta alba* (L.) Hassak, Family: Asteraceae) is commonly known as False daisy or Ink plant in English and locally known as *Bhringraj*, *Bhumiraj*, *Aali jhar*, and *Nash jhar* in Nepali language (Figure 1) [7,8]. *E. prostrata* is a medium-sized, branched, annual herb-bearing white flower natively found in the tropical and subtropical regions of the world [9,10]. It grows mostly in moist sites such as swamp edges, river or lake banks and edge of rice-fields and easily propagated and spread throughout China, India, Nepal, Brazil and other parts of the world [8,11,12,13]. It is widely distributed in tropical and sub-tropical regions of Asia, Africa, and South America (Figure 2) [14,15]. Traditionally, it is used to treat different skin problems such as wounds, hair loss prevention, and dermatitis. The leaves are used to treat snakebite in India, China, and Brazil. The mixture of leaf juice and honey is used to cure catarrh in infants [16,17]. The juice of *E. prostrata* is taken orally or applied locally to promote hair growth [18]. 

Various research articles have been published regarding the chemical constituents and biological activities of different plant parts of *E. prostrata.* Critical analysis of these published scientific studies would provide the detailed understanding about the potential use of *E. prostrata* as medicine, cosmetic, and other formulations along with highlighting the gaps in research. Hence, the main aim of this article was to collect the information about traditional uses, chemical constituents, and the biological activities of *E. prostrata.*

## 2. Methods

The scientific information on *E. prostrata* was retrieved from various online bibliographic databases such as ScienceDirect, PubMed, Google scholar, SciFinder, etc. and books and proceedings. The articles with rigorous quality were selected for the review. Relevant articles published before June 2021 were collected using the key words *Eclipta prostrata*, *Eclipta alba*, phytochemistry, traditional use, biological activity, pharmacological activity, etc.

## 3. Ethnomedicinal Uses

This plant is widely used in different regions of India for the treatment of skin problems, hepatic problems such as jaundice, gastrointestinal problems, respiratory problems such as asthma, and other symptoms such as fever, hair loss and whitening of hair, cuts, and wounds, spleen enlargement, etc. [19,20]. The leaf juice is used with honey to cure catarrh in infants, shoot juice and mustard oil is taken together for diarrhea and dysentery, and the whole plant is used for the treatment of symptoms related to hepatitis, itching, hemoptysis, bleeding, hematuria, diarrhea, and diphtheria [16]. The leaves and shoots are used in preventing infection in wounds and its treatment in Nepal [7,8,11,21]. Some ethnic groups in South American countries use it to treat snakebites [22]. In Ayurveda, it is used for its revitalizing and anti-aging properties [23]. Many ethnic groups of Bangladesh use it for the treatment of jaundice [24,25]. The plant juice has been used to control, kill, and inhibit the growth of diseases carrying vectors such as mosquito [26,27]. Additionally, it is also used to treat different types of symptoms such as acidity, alopecia [28], gingivitis, fever, body pain, asthma, bronchitis, burns, constipation, wounds, wrinkles, edema, pimples, and other skin diseases [29,30,31,32]. 

## 4. Bioactive Chemical Constituents

**Figure 3:**
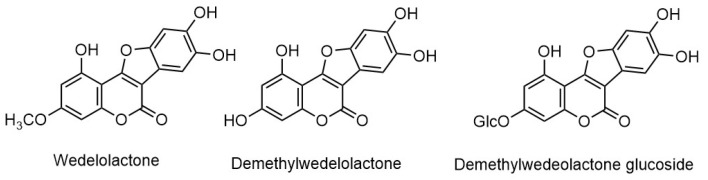
Structures of major coumestan derivatives.

**Figure 4:**
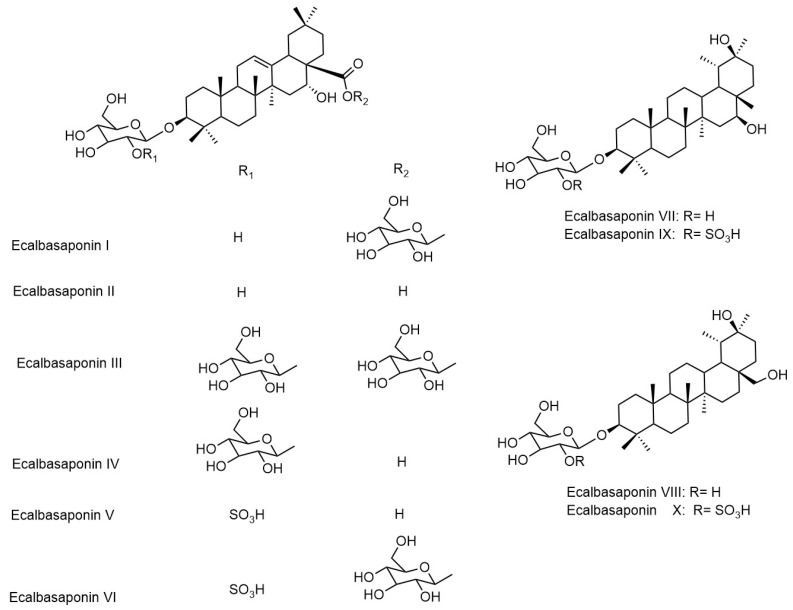
Structures of main triterpene saponins.

**Figure 5:**
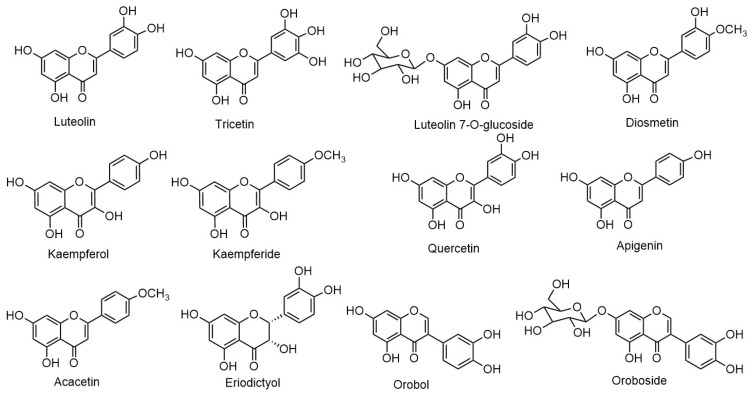
Structures of main flavonoids.

*Eclipta prostrata* contains a wide range of active phytoconstituents, which includes coumestan derivatives, triterpene saponins, steroidal saponins, triterpenes, steroids, steroidal alkaloids, flavonoids, phenolic acids, thiophene derivatives and many other compounds. Most of the chemical analysis are reported for whole plant or aerial parts. The detailed list of these compounds is given in Table 1 and Table 2. The structures of main coumestan derivatives, triterpene saponins and flavonoids are represented in Figure 3, Figure 4 and Figure 5, respectively. 

## 5. Biological Activities

Due to the wide range of ethnomedicinal values and applications, several studies have been performed regarding the biological activities of extracts and compounds obtained from *E. prostrata* using both in vitro and in vivo models [59,60,61]. Some of them are discussed in detail in following sub-sections.

### 5.1. Antioxidant Activity

The antioxidant effects of *E.* *prostrata* were evaluated in Charles River Sprague-Dawley rats. The extract at 50 mg/kg and 100 mg/kg dose significantly reduced the oxidative biomarkers such as serum lipid peroxide, serum hydroxyl radical levels in [41]. 

In another study, the in vitro antioxidant activity was evaluated based on the 1,1-diphenyl-2-picrylhydrazyl (DPPH) free radical scavenging assay. An IC_50_ value of extract was determined to be 45.68 µg/mL for the whole plant as compared to the IC_50_ of 3.26 µg/mL of standard ascorbic acid. When evaluated using hydrogen peroxide scavenging assay, the extract showed potent activity with the IC_50_ values of 1.34 µg/mL as compared to ascorbic acid (IC_50_: 1.03 µg/mL) [9]. The antioxidants present in the extract of *E. prostrata* showed the reduction of ferricyanide complex (Fe^+3^) to ferrous form (Fe^+2^) in a dose-dependent manner. The highest reducing ability (75.59%) for the whole plant of *E. prostrata* was reported at 250 µg/mL concentration. The IC_50_ values for reducing ability of the extract was 100 µg/mL [62]. The studies are mostly conducted using in vitro methods and detailed mechanism is yet to be established.

### 5.2. Antimicrobial Activity

The butanol and water extract, at a concentration of 3 mg/disc inhibited the growth of *Bacillus cereus* by 45% and 42%, respectively. The highest growth inhibition of 63% for butanol extract and 54% for ethyl acetate fraction were reported against *B. subtilis* at the concentrations of 3 mg/disc. The butanol extracted sample showed inhibition of 57%, 72%, and 89% at concentrations of 1, 2 and 3 mg/disc, respectively and that of ethyl acetate extract showed the growth inhibitions of 40, 57, and 83%, at concentrations of 1, 2, and 3 mg/disc, respectively. Similarly, methanol extract inhibited the growth of *Candida albicans* by 38, 48, and 59% at concentrations of 1, 2, and 3 mg/disc, respectively. [16].

A coumestan derivative, wedelolactone (10 µg/mL), isolated from the plant showed promising antibacterial properties against *Staphylococcus epidermidis*, *Salmonella* Typhimurium, *Staphylococcus aureus*, *Pseudomonas aeruginosa*, *Shigella flexneri* and *Escherichia coli* with the zone of inhibition (ZOI) of 10.24, 9.16, 9.14, 8.0, 7.60, 8.60 mm, respectively at 10 µg/mL and minimum inhibitory concentration (MIC) of 15, 25, 20, 1250, 1300, 1000 µg/mL respectively [21]. 

The percentage inhibition of 36.84, 38.94, and 47.36% was reported against *Aspergillus niger* by 2 mg, 3 mg, and 4 mg of ethanolic extract of *E. prostrata,* respectively as compared to the inhibition of 64.21% by 100 µg of standard fluconazole. Similarly, the percentage inhibitions of 20, and 28% were reported against *Aspergillus ustus* by 3 mg, and 4 mg of ethanolic extract, respectively as compared to inhibition of 40% by 100 µg of standard fluconazole. Similarly, against *Aspergillus ochraceus,* the percentage inhibition of 41.37, 44.82, and 51.52% in was reported for 2 mg, 3 mg, and 4 mg of ethanolic extract, respectively compared to the inhibition of 37.93% by 100 µg of standard fluconazole [9]. 

The alkaloids from the leaves were also studied for the antimicrobial properties against *E. coli*, *P. aeruginosa*, *Shigella boydii*, *S. aureus* and *S. faecalis* by agar-well diffusion and broth-microdilution methods. The ZOI ranged from 9.8–16.5 mm in 500 μg/mL and MIC ranged from 42–89 μg/mL for the sample which was found to be comparable to the positive control ciprofloxacin with MIC range of 0.8–1.3 μg/mL [63].

### 5.3. Hepatoprotective Activities

In vivo hepatoprotective activity was evaluated by Thirumalai et al. [64]. The aqueous extract of leaves of *E. prostrata* was administered to carbon tetrachloride (CCl_4_)-induced hepatotoxicity in male albino rats. The extract (250 mg/kg b.w.) reduced the elevated levels of all the biochemical parameters such as glutamic-oxaloacetic transaminase (GOT), glutamine-pyruvate transaminase (GPT) and bilirubin.

The chloroform extract of the roots and methanol extract of the leaves of *E. prostrata* were investigated for hepatoprotective activity in CCl_4_-induced hepatotoxicity in male albino rats by measuring the levels of lysosomal enzymes. The chloroform extract of roots showed 47.96% reduction in the lysosomal enzyme whereas, methanol extract of leaves showed a 72.8% reduction. The triterpenoid and alkaloid fractions obtained from the methanol extract of showed the 78.78% and 60.65% reduction in lysosomal enzyme levels, respectively. Similarly, triterpenoid saponin fraction and coumestan fractions obtained from the chloroform extract of roots reduced the lysosomal enzyme level by 52.41% and 75.6%, respectively [65]. 

The ethanolic extract (50%) obtained from the whole plant of *E. prostrata* was studied for its hepatoprotective effect. The study was conducted in rats against CCl_4_-induced hepatic damage. The result revealed that *E. prostrata* extract significantly normalized the biochemical parameters by counteracting the hepatic drug-metabolizing enzyme inhibition. The reduction of a biochemical parameter such as hepatic lysosomal acid phosphatase and alkaline phosphatase by CCl_4_ was observed to be restored when treated with *E. prostrata*. This study shows that the hepatoprotective activity of this plant relies on the regulation of the levels of hepatic microsomal drug-metabolizing enzymes [66]. The echniocystic acid and eclabasaponin II from the aerial parts showed antiproliferative activity in hepatic stellate cells [67]. 

The experiments were performed mostly in vivo using only rats. Its clinical significance is not well studied. Some studies lack positive and negative controls. Further, studies on human and animal models along with the isolation of active compounds from this plant may lead to the discovery of potent therapeutic agents.

### 5.4. Anti Hyperlipidemic Activities

Diacylglycerol acyltransferase (DGAT) is a key enzyme of biosynthesis in the final step of the glycerol phosphate pathway. The triglycerides synthesized in excess causes various symptoms such as type II diabetes mellitus, hypertriglyceridemia, and obesity. The active polyacetylene constituents from the stem of *E. prostrata* was tested for the inhibition of the DGAT enzyme. Kuraridine was used as positive control, which is known as DGAT inhibitor. A total of 8 isolates showed potent activity and IC_50_ values was of 74.4 ± 1.3 to 101.1 ± 1.1 μM range while that of positive control was 10.4 ± 1.4 μM [54].

The effect of methanol extract of *E. prostrata* on non-alcoholic fatty liver in rats was evaluated after inducing fatty liver via high-fat diets with cholesterol and cholic acids. The biochemical and histopathological analysis revealed that high dose treatment of *E. prostrata* (200 mg/kg and 300 mg/kg) exhibited significant improvement in lipid profile and liver function [68]. 

### 5.5. Cerebroprotective and Nervous System Related Activities

The hydroalcoholic extract of *E. prostrata* was subjected to the study of cerebroprotective function in Wister albino rats [12]. The pretreatment of the hydroalcoholic extract global cerebral ischemia model resulted in a great difference in ischemia control. *E. prostrata* extract (250 and 500 mg/kg) administration gradually improved the antioxidant enzyme levels, decreased brain edema, and altered some histopathological status in mice after bilateral cerebral artery occlusion.

The aqueous extract obtained from leaves of *E. prostrata* at the dose of 100 and 200 mg/kg was evaluated for its potential application on transfer latency (TL) as a parameter of acquisition and retrieval learning in rats using an elevated plus-maze. The administration of extract was reported to significantly improve the retrieval memory [69]. 

The acetylcholine formation in the brain and oxidative stress inhibition in the brain and serum of rats were accessed before and after feeding the experimental diet. The rats were fed with 25, 50,100 mg/kg of butanol fraction of the aqueous extract of *E. prostrata*. The acetylcholine level was increased by 9.6–12.1% in 50 mg/kg and 100 mg/kg fed group as compared to control. Monoamine oxidase-B activity and superoxide radical levels were decreased by 10.5% and 9.4%, respectively in the 100 mg/kg treated group [70].

The in vivo anti-epileptic activities were studied by Tambe et al. in mice [71]. This study isolated the luteolin as a major constituent from the leaves of *E. prostrata* which was evaluated for its anticonvulsant and anti-epileptic activities. The study found that luteolin exhibited anticonvulsant activities and also ameliorated the oxidative level in the mice induced by kindling.

### 5.6. Anti-Diabetic/Anti-Hyperglycemic Activities

The in vitro α-amylase inhibition activity of methanol extract of the whole plant of *E. prostrata* was evaluated. The result revealed mild potency in α-amylase inhibition indicating the potential anti-diabetic property with the IC_50_ value of 322.138 ± 0.025 µg/mL [72]. 

In another experiment, the rats were injected with streptozotocin in the peritoneum at a dosage of 70 mg/kg of body weight to induce diabetics. With the administration of wedelolactone from *E. prostrata* to diabetic rats, HbA1c (%) level was recovered from 10.3 ± 0.72 in the untreated diabetic rats to 7.2 ± 0.52 in wedelolactone treated diabetic group. The change in a biochemical parameter such as an increase in urea and creatinine in the streptozotocin treated group was declined towards normal by treating with wedelolactone. The c-peptide and insulin secreted from β-cells were investigated to confirm the treatment by wedelolactone and these hepatic-parameters were shifted towards the normal values after 28 days of treatment of wedelolactone [73].

The ethanolic extract of *E. prostrata* showed inhibition of α-glucosidase in a dose-dependent manner. About 85% inhibition was observed at 100 µg/mL concentration while that of standard acarbose showed around 56% inhibitory at the same concentration. The IC_50_ value of the extract was 54 µg/mL. The aldose reductase activity was also found to be inhibited in a dose-dependent manner. The maximum inhibition was observed at the concentration of 10 µg/mL at which the enzyme activity was decreased by 88.6%. The IC_50_ values of the extract were calculated to be 4.5 µg/mL [74].

Many polyherbal formulations include *E. prostrata* as an essential ingredient. It is reported to act upon the pancreas via restoration and regeneration of β-cell and to possess antidiabetic activity [75]. 

The antidiabetic properties of *E. prostrata* in alloxan-induced diabetic rats were evaluated using the leaf suspension of the plant (2 & 4 g/kg) orally. The extract significantly normalized the biochemical parameters altered by diabetes. The activity of liver hexokinase was increased; blood glucose level and glycosylated hemoglobin were reduced due to the reduced activity of glucose-6 phosphatase and fructose-1,6-bisphosphatase which revealed the anti-hyperglycemic activity of *E. prostrata* in the rats [76]. However, the mechanism on the chemical model related to the antidiabetic properties has not been well studied.

### 5.7. Anticancer Activities

The methanol extract of *E. prostrata* was administered orally at the dose of 250 and 500 mg/kg to Ehrlich ascites carcinoma (EAC) bearing mice and it was found to increase life span. It also decreased the viable cell count and tumor volume of the tumor-bearing mice when compared to that of control. As compared to EAC control, *E. prostrata* extract restored the hematological parameters such as red blood cells (RBC) count and hemoglobin content. In the treated group, the percentage of lymphocytes was increased with a decreased level of neutrophils [77].

The in vitro and in vivo tumor growth inhibition of breast cancer cells by the chloroform fraction of methanol extract of *E. prostrata* was reported by Arya et al. [78] which resulted the marked inhibition of the breast tumor growth in vitro and in vivo by selective regulation of Hsp60 cell. The extract specifically activated the apoptotic pathway by the process of disruptions of mitochondrial membrane potential by upregulating and downregulating the Hsp60 and anti-apoptotic protein XIAP, respectively. The extract was also found to mitigate tumor-induced hepato-renal toxicity. Further, the LC-MS approach identified luteolin as a major contributor to the anti-cancer activities.

The antitumor activities of terthiophenes isolated from the n-hexane fraction of *E. prostrata* were evaluated against the endometrial cancer cells (Hec1A and Ishikawa cells) by 3-(4,5-dimethylthiazol-2-yl)-2,5-diphenyltetrazolium bromide (MTT) assay. Among the five terthiophenes isolated, α-terthienylmethanol was found to be most potent to inhibit the Hec1 (IC_50_ = 0.38) and Ischikawa (IC_50_ = 0.35) cancer cells [56].

The cytotoxic activities of various compounds isolated from aerial parts of *E. prostrata* were evaluated against human ovarian cancer cell lines SKOV3. Cytotoxicity was assessed by MTT assay, and the IC_50_ values of more than ten compounds were less than 100 µM and that of positive control cisplatin was 11.25 ± 0.27 µM [79].

The antiproliferative potential of ethanolic extract of *E. prostrata* was evaluated using MTT assay in which the extract inhibited the cell proliferation in a dose-dependent manner. The IC_50_ value for MTT assay was calculated from the growth curve and was found to be 22.1 ± 2.9 g/mL for HepG2, 25.3 ± 3.6 g/mL for A498, and 50.2 ± 8.7 g/mL for C6 cell lines. This result showed that extract was much effective for HepG2 cells. When treated with different doses of the extract resulted in a decrease in cell density which was supported by the MTT assay. The cells in all three cell lines were found to be detached, round, and floating at higher concentrations of extract [80]. However, the effects on the biochemical pathway of healthy cells have not been well studied yet.

### 5.8. Hair Growth Promoting Activity

The dorsal area was shaved and the effect on growth of hair was observed in Wister rats by Mondal et al. to evaluate hair growth promoting activity [81]. The time for the hair growth initiation was noted in days and the time required to complete the hair growth was 19.01 ± 0.51 days for ethanolic extract of the leaves of *E. prostrata* treated group compared to the time taken for 2% minoxidil (standard) treated group (16.05 ± 0.41 days). The hair growth in the extract-treated group observed at the 10th, 20th, and 30th day was 8.91 ± 0.03 mm, 16.01 ± 0.01 mm, and 21.08 ± 0.03 mm, respectively as compared to the length of hair increased in 2% minoxidil (standard) treated group as 9.23 ± 0.01 mm, 17.63 ± 0.02 mm and 22.13 ± 0.04 mm, respectively. Both the extract and standard treated groups showed a significant increase in as compared to the control group.

The effects of petroleum ether extract of *E. prostrata* on stimulation of the hair coverage area of the nude mice were evaluated after applying specific concentrations of petroleum ether extract vs. the vehicle and or 2% minoxidil. A score of 0 to 8 was given for each mouse to estimate the effects of petroleum ether extract on the hair coverage area of the mice in all treatment groups. From day 8 the mice treated with petroleum ether extract of *E. prostrata* showed maximum hair growth than in the mice of other groups and gradually covered the maximum area of the body on day 16. But rapid hair loss was observed in the minoxidil and other treatment groups in the case of the nude mice. In terms of hair density, the mice treated with petroleum ether extract exhibited a significant increase in hair density compared to the other groups on day 8 and 16. Although minoxidil had a significant effect on sustaining hair density on day 8, progressive hair loss occurred and hair density also decreased on day 16 [82].

The hair growth cycle was found to be significantly affected by minoxidil and petroleum ether extract of *E. prostrata* treatment. In the case of control, one to two hair follicles were in the catagenic phase, while most were in the telogen phase. A similar scenario was observed in the group treated by ethanolic extract with the absence of anagen hair follicle. The reverse scenario was observed in minoxidil and petroleum ether extract treated group; where most hair follicles were in anagenic phase, a few hair follicles in catagen phase and almost no hair follicles were in telogen phase. In the control group, there were low anagenic hair follicles, but in the case of the 2% and 5% petroleum ether extract group, it was about 68 ± 1.2% and 70 ± 1.6% anagenic hair follicles, respectively. The major contributors were wedelolactone and β- sitosterol in petroleum ether extract, which were responsible for hair growth promotion [28].

The study on hair growth promotion by a polyherbal formulation containing *E. prostrata* was reported by Roy et al. [83]. The time taken for complete hair growth was 18 d in the group receiving oil of 10% *E. prostrata,* 10% hibiscus, and 5% jatamasi (DF3) and 22 d in the group receiving 10% *E. prostrata*, 5% hibiscus and 10% jatamasi (DF2). On comparing the activity of DF3 and minoxidil, DF3 hair oil formulation showed a better result of hair growth. Mean hair length was 4.6 mm and 3.6 mm in DF3 and DF2 groups, respectively. The study revealed the fact that DF3 formulation resulted in significant increment in the number of hair follicles in the anagen phase of the hair growth cycle. The percentage of the population of anagen follicle was 67 in the standard group, while in DF3 and DF2 formulations, it was 82 and 65, respectively. The results revealed that DF3 formulation had more potent to hair growth activity [83]. However, the role of *E. prostrata* in the treatment of hair fall caused by other reasons such as diseases, aging, and genetics are not clear yet.

### 5.9. Immunomodulatory Activities

The immunostimulatory effects of *E. prostrata* in tilapia fish (*Oreochromis mossambicus*) was studied. A diet with 0.01, 0.1, and 1% of the aqueous extract of *E. prostrata* were fed to fish, and after successive weeks, various nonspecific humoral responses (complement, antiprotease, and lysozyme), cellular responses (reactive oxygen and nitrogen production, myeloperoxidase content) and disease resistance were observed against the activity of common pathogen of fish and human, *Aeromonas hydrophila*. After feeding aqueous extract for 1, 2, or 3 weeks, the activity of lysozyme was increased significantly. After 1 week of aqueous extract supplement, the enhancement of reactive oxygen species production and myeloperoxidase content was observed. The mortality rate in fish had decreased significantly when fed with the extract [84].

The wedelolactone obtained from the methanol extract of the whole plant of *E. prostrata* was reported to show immunomodulatory responses in mice at different dose ranges from 100 to 500 mg/kg. Various parameters such as carbon clearance, antibody titer, and cyclophosphamide immunosuppression were accessed, which showed the significant increase in the phagocytic index and antibody titer which resulted in a significant ratio of the phagocytic index and white blood cells (WBC) count [17].

### 5.10. Others Activities

The study using in vitro assay suggested that wedelolactone from *E. prostrata* extract exhibited anti-hepatitis C virus (HCV) activity. It was reported that the mechanism was due to the inhibition of HCV replicase activity in the cell culture systems treated with wedelolactone [33].

*E. prostrata* was reported to exhibit active inhibition activity against *Bothrops jararacussu* venom. The root extract was reported to have inhibition of the phospholipase A2 activity nearly by 50%. The highest result was obtained for the extract of aerial part as compared to other extracts, which showed approximately 70% inhibition of the phospholipase A2 activity [22].

Echniocystic acid, the triterpene component of *E. prostrata* was found to be effective in treatment of ovariectomy-induced osteoporosis in rats. Administration of 5–15 mg/kg per day for 12 weeks was reported to prevent the level of stress and Young’s modulus of the femur. The compound also restored the bone biomarkers level such as osteocalcin, alkaline phosphatase, deoxypyridinoline, phosphorus, and urinary calcium. Treatment from *E. prostrata* also prevented the altercation of the bone mineral density, improved trabecular architecture, trabecular number, and trabecular thickness [85]. 

The methanol extract of leaves of *E. prostrata* was reported to show nephroprotective activity in gentamycin-induced nephrotoxicity in rats. The activity of the extract was evaluated for its ability to decrease the gentamicin-induced elevations of biochemical parameter such as serum creatinine and histological changes in renal tissues. It was reported that the extract of could significantly act as a nephroprotective agent against gentamicin toxicity comparable to quercetin [86]. Similar studies were made on doxorubicin hydrochloride-induced nephrotic syndrome on mice. The significant improvement on biochemical parameter such as urine protein, triglyceride etc. was seen on the *E. prostrata* treated group. The pathological changes in kidney also supported the fact [87]. Another study revealed that the major constituents of *E. prostrata* i.e., wedelolactone could inhibit the abnormal proliferation of human renal mesangial cells (HRMCs) due to inflammation of renal tissues via the regulation of NF-κB signaling pathway [88].

## 6. Toxicity Evaluation against Brine Shrimp and Mosquito

Uddin et al. evaluated toxicity of the ethanol extract of *E. prostrata* by brine shrimp lethality assay. In this assay, the concentrations of 25, 31.25, 62.5, 125, 250, 500 µg/mL of extract was used to determine cytotoxicity against brine shrimp. The percentage lethality was 0, 10.0, 41.70, 61.70, 83.30, and 100% for the concentrations of 25, 31.25, 62.5, 125, 250, 500 µg/mL of extract, respectively. The lethality was observed in a dose-dependent manner [9].

The toxicity of crude extracts of *E. prostrata* extracted in different solvent was tested against *Aedes aegypti*. The lethality values were expressed in LC_50_ and LC_90_ values. The LC_50_ values of hexane, benzene, ethyl acetate, methanol, chloroform and methanol extracts of *E. prostrata* against larvae at the early stage were 151.38, 165.10, 151.38, 154.88, 146.28, and 127.64 ppm, while LC_90_ values were, 297.70, 274.34, 288.61, 274.42, and 245.73 ppm, respectively. The methanol extract was reported to have highest larvicidal activities followed by other solvents such as chloroform, benzene, ethyl acetate, and hexane extract. The relation of egg hatchability and that of concentration of leaf extract of *E. prostrata* were estimated. The methanol extract exerted 0% hatchability (100% mortality) at 300 ppm [26].

## 7. Conclusions

*Eclipta prostrata* is widely used as traditional medicine in various countries specially for skin, liver and stomach problems, and for promoting hair growth. Various compounds such as coumestan derivatives, steroidal and triterpenoid saponins, phenolic acids, flavonoids, and substituted thiophenes were isolated and identified from the extracts. Similarly, various biological activity evaluations were performed for extracts and isolated compounds such as antioxidative, antimicrobial, hepatoprotective, anticancer, hair growth promoting activities. Many of these activities were performed based on in vitro methods and mechanisms of action are not explored in detail using animal models. Properly designed clinical studies are necessary to evaluate the safety and efficacy *E. prostrata* in future.

## Figures and Tables

**Table 1:** Coumestan, steroid and triterpene derivatives and related compounds from various parts of *E. prostrata*.

Chemical Compounds (Class/Constituents)	Plant Parts	References
***Coumestan derivatives***		
Wedelolactone	Leaves	[21,33,34,35,36]
Demethylwedelolactone	Leaves	[35,36]
Isodemethylwedelolactone	Whole plant	[36]
Strychnolactone	Whole plant	[36]
Demethylwedelolactone glucoside	Aerial Parts	[37]
***Steroidal and triterpene saponins, steroidal alkaloids, steroids and triterpenoids***		
Eclalbasaponins I	Whole plant	[34,38,39]
Eclalbasaponins II	Whole plant	[34,38,39]
Eclalbasaponins III	Whole plant	[34,38,39]
Eclalbasaponins IV	Whole plant	[38,39]
Eclalbasaponins V	Whole plant	[34,38,39]
Eclalbasaponins VI	Whole plant	[38,39]
Eclalbasaponins VII	Whole plant	[38]
Eclalbasaponins VIII	Whole plant	[38]
Eclalbasaponins IX	Whole plant	[38]
Eclalbasaponins X	Whole plant	[38]
Eclalbasaponin XI	Whole plant	[38]
Eclalbasaponin XII	Whole plant	[38]
Eclalbasaponin XIII	Whole plant	[38]
Eclalbasaponin A	Whole plant	[40]
Eclalbasaponin B	Whole plant	[40]
Eclalbasaponin C	Whole plant	[40]
Eclalbasaponin D	Whole plant	[40]
Echinocystic acid	Whole plant	[40]
Echinocystic acid-3-*O*-(6-*O*-acetyl)-*β*-D-glucopyranoside	Aerial parts	[41]
Eclalbatin	Aerial Parts	[42,43]
3*β*,25-Dihydroxy-23*E*-lemmaphyll-8,23-diene	Whole plant	[44]
16*α*-Hydroxy-olean-12-en-3-on-28,21*β*-olide	Whole plant	[44]
3*β*-Hydroxy-17-epi-28-norolean-12-en-16-one 3-*O*-*β*-D-glucopyranoside	Whole plant	[44]
3*β*-*O*-(6-*O*-Crotonyl-*β*-D-glucopyranosyl)-16α-hydroxy-olean-12-en-28-oic acid 28-*O*-*β*-D-glucopyranosyl ester	Whole plant	[44]
3-*O*-(2-*O*-Acetyl-*β*-D-glucopyranosyl) oleanolic acid-28-*O*-(*β*-D-glucopyranosyl) ester	Aerial parts	[45]
3-*O*-(6-*O*-Acetyl-*β*-D-glucopyranosyl) oleanolic acid-28-*O*-(*β*-D-glucopyranosyl) ester	Aerial parts	[45]
3-*O*-(*β*-D-Glucopyranosyl) oleanolic acid-28-*O*-(6-*O*-acetyl-*β*-D-glucopyranosyl) ester	Aerial parts	[45]
3*β*,16*β*,29-Trihydroxy oleanane-12-ene-3-*O*-*β*-D-glucopyranoside	Aerial parts	[46]
3,28-di-O-*β*-D-Glucopyranosyl-3*β*,16*β*-dihydroxy oleanane-12-ene-28-oleanlic acid	Aerial parts	[46]
3-*O*-*β*-D-Glucopyranosyl-(1-2)-*β*-D-glucopyranosyl oleanlic-18- ene acid-28-*O*-*β*-D-glucopyranoside	Aerial parts	[46]
(*20S*)(*25S*)-22,26-Imino-cholesta-5,22(*N*)-dien-3*β*-ol (Verazine)	Leaves	[47]
20-*epi*-3-Dehydroxy-3-oxo-5,6-dihydro-4,5-dehydroverazine	Leaves	[32,47]
(*20R*)-20-Pyridyl-cholesta-5-ene-3*β*,23-diol (Ecliptalbine)	Leaves	[47]
(*20R*)-25*β*-Hydroxyverazine	Leaves	[47]
20-*epi*-4*β*-Hydroxyverazine	Leaves	[47]
20-*epi*-25*β*-Hydroxyverazin	Leaves	[47]
4*β*-Hydroxyverazine	Leaves	[47]
25*β*-Hydroxyverazine	Leaves	[47]
Lanost-5,24-dien-3*β*-ol-18, 21-olide -3*β*- yl tetradecanoate	Whole plant	[48]
α-Amyrin	Whole plant	[43]
Ursolic acid	Whole plant	[43]
Oleanolic acid	Whole plant	[43]
3-Oxo-16*α*-hydroxy-olean-12-en-28-oic acid	Aerial parts	[49]
Machaeroceric acid	Aerial parts	[34]
Silphioside C	Whole plant	[50]
*β*-Sitosterol	Whole plant	[36]
Stigmasterol	Leaves/Stems	[40]
Stigmasterol-3-*O*-glucoside	Aerial parts/leaves/Stems	[19,34,40]
3-*O*-(6′-*O*-Palmitoyl-*β*-D-glucopyranosyl) stigmasterol	Whole plant	[50]
Daucosterol	Leaves/Stems	[40]

**Table 2:** Flavonoids, phenolic acids, substituted thiophines, and other compounds present in the *E. prostrata*.

***Flavonoids***		
Luteolin	Aerial parts	[34,41,51]
Tricetin	Aerial parts	[34]
Luteolin-7-*O*-*β*-D-glucoside	Aerial parts	[34,41,51]
Diosmetin	Aerial parts	[52]
Skullcapflavone Ⅱ	Whole plant	[51]
Kaempferol	Whole plant	[51]
Kaempferol-7-*O*-*α*-D-rhamnoside	Aerial parts	[34]
Kaempferide	Whole plant	[51]
Quercetin	Aerial parts	[51,53]
Quercetin-3-*O*-*β*-D-glucoside	Aerial parts	[34]
Apigenin	Aerial parts	[34,41,51]
Acacetin	Whole plant	[51]
Acacetin-7-*O*-rutinoside	Whole plant	[51]
Eriodictyol	Whole plant	[50]
Pyracanthoside	Whole plant	[50]
Hesperetin-7-*O*-*β*-D-glucoside	Aerial parts	[34]
3′-Hydroxybiochanin A	Aerial Parts	[37,49]
Orobol (isoluteolin)	Whole plant	[31,34]
7-*O*-Methylorobol-4′*-O*-*β*-D-glucopyranoside	Aerial Parts	[34,49,50]
7-Dihydroxyl-3′, 6′-dimethoxylisoflavone-7-*O*-glucoside	Whole plant	[51]
3′-*O*-Methylorobol	Aerial parts	[50,52]
Pratensein	Aerial parts	[37,49,50]
Pratensein-7-*O*-*β*-D-glucopyranoside	Aerial parts	[41,50]
Oroboside (Orobol-7-O-β-D-glucoside)	Whole plant	[34,37,50,51]
***Phenolic acids***		
Protocatechuic acid	Leaves/Steam/Whole plant	[34,36,40,43]
4-Hydroxybenzoic acid	Leaves/Steam	[34,40,43]
Vanillic acid	Aerial parts	[34]
Syringic acid	Aerial parts	[34]
Chlorogenic acid	Aerial parts	[34]
Syringic acid	Aerial parts	[34]
Tachinoside	Whole plant	[50]
Coniferylaldehyde	Whole plant	[50]
Leonuriside A	Whole plant	[50]
Caffeic acid	Whole plant	[50]
Ferulic acid ethyl ester	Whole plant	[50]
Caffeic acid ethyl ester	Whole plant	[50]
***Lignin***		
Ecliptalignin A		
***Coumarins***		
Psoralen	Whole plant	[51]
Isopsoralen	Whole plant	[51]
***Polyacetylinic compounds***		
(5*E*)-Hendeca-1,5- dien-7,9-diyne-diol-4-O-*β*-D-glucopyranoside	Stem	[54]
(5*E*)-Trideca-1,5-dien-7,9,11-triyne-3,4-diol-4-*O*-*β*-D-glucopyranoside	Stem	[46,54]
3-*O*-*β*-D-Glucopyranosyl1-hydroxy-4*E*,6*E*-tetradecene,8,10,12-triyne	Stem	[46,54]
2-*O*-*β*-D-Glucosyltrideca-3*E*,11*E*-dien5,7,9-triyne-1,2,13-triol	Stem	[54]
2-*O*-*β*-D-Glucosyltrideca-3*E*,11*E*-dien-5,7,9-triyne-1,2-diol	Stem	[54]
2-*O*-*β*-D-Glucosyltrideca-3*E*,11*Z*-dien-5,7,9-triyne3–1,2-diol	Stem	[54]
***Substituted thiophenes***		
5-Hydroxymethyl-(2,2′:5′,2″)-terthienyl tiglate	Whole plant	[55]
5-Hydroxymethyl-(2,2′:5′,2″)-terthienyl agelate	Whole plant	[55]
5-Hydroxymethyl-(2,2′:5′,2″)-terthienyl acetate	Whole plant	[55]
5-Formyl-(2, 2:5, 2″)-terthiophene (Ecliptal)	Whole plant	[56]
5-Hydroxymethyl-(2, 2: 5, 2″)-terthiophene (α-terthienylmethanol)	Whole plant	[56]
5-Methoxy-(2, 2:5, 2″)-terthiophene	Whole plant	[56]
3′-Methoxy-2,2′:5′,2″-terthiophene	Aerial parts	[41]
5-(3″,4″-Dihydroxy-1″-butynyl)-2,2′-bithiophene	Aerial parts	[41]
α-Terthienyl	Aerial parts	[41]
α-Formylterthienyl	Whole plant	[54]
α-Terthienyl methanol	Whole plant	[41,54,56]
3′-Methoxy-2,2′:5′,2″-terthiophene	Aerial parts	[41]
4-(2,2′-Bithiophen-5-yl)but-3-yne-1,2-diol	Aerial parts	[57]
Arctinol B	Aerial parts	[57]
2-(Penta-1,3-diynyl)-5-(3,4-dihydroxy-but-1-ynyl)-thiophene	Aerial parts	[57]
6-Methoxy-arctinol-b	Aerial parts	[57]
5-[l-(4-Hydroxybut-l-ynyl)]-2,20 -bithiophene-50 -carbaldehyde	Aerial parts	[57]
5-Hydroxymethyl- (2,2′:5′,2′’-terthienyl)	Aerial parts	[57]
5′-Hydroxymethyl-5-(3-butene-1-ynyl)-2,2′ -bithiophene	Aerial parts	[46,57]
3′-Hydroxy-2,2′:5′,2′’ terthiophene-3′-O-*β*-D-glucopyranoside	Aerial parts	[57]
Ecliprostin A	Aerial parts	[58]
Ecliprostin B	Aerial parts	[58]
Ecliprostin C	Aerial parts	[58]
***Alkaloids***		
Crinumaquine	Whole plant	[51]
2,3,9,12-Tetramethoxyprotoberberine	Whole plant	[51]
***Lignans***		
Pinoresinol-4-O-β-D-glucopyranoside	Whole plant	[50]
4,4′-Dimethoxy-3′-hydroxy-7,9′:7′,9-diepoxylignan-3-O-β-D-glucopyranoside	Whole plant	[50]
Syringaresinol-4′-O-β-D-glucopyroside	Whole plant	[50]
Lanicepside A	Whole plant	[50]
Longifloroside	Whole plant	[50]
***Other compounds***		
1-*O*-Octadecanoyl-2-*O*-(9*Z*,12*Z*-octadecadienoyl)-3-*O*-[*α*-D-galactopyranosyl- (1′′→6′)-*O*-*β*-D-galactopyranosyl]glycerol	Whole plant	[50]
(*2S*)-3-*O*-α-D-Galactopyranosyl-(1′′→6′)-*β*-D-galactopyranosyl-1,2-di-*O*-[(9Z,12Z,15Z)-octadeca-9,12,15-trienoyl]-sn-glycerol	Whole plant	[50]
1-*O*-(9*Z*,12*Z*,15*Z*-Octadecatrienoyl)-2-*O*-hexadecanoyl-3-*O*-[α-D-galactopyranosyl-(1′′→6′)-*O*-*β*-D-galactopyranosyl]glycerol	Whole plant	[50]
1-*O*-(*β*-D-glucopyranosyl)- (*2S,3S,4R,8Z*)-2*N*-[(2′*R*)-2′-hydroxytetracosanoyl]-8-(*Z*)-octadecene-1,3,4-triol	Whole plant	[50]
(*2S,3S,4R,10E*)-2-[(*2′R*)-2′- Hydroxytetracosanoylamino]-10-octadecene-1,3,4-triol	Whole plant	[50]
(*3S,5R,6S,7E,9R*)-3-Hydroxy-5,6-epoxy-*β*-ionyl-3-*O*-*β*-D-glucopyranoside	Whole plant	[50]
Euodionoside A	Whole plant	[50]
Junipeionoloside	Whole plant	[50]
Calaliukiuenoside	Whole plant	[50]
*rel*-(*1S,2S,3S,4R,6R*)-1,6-Epoxy-menthane-2,3-diol-3-*O*-*β*-D-glucopyranoside	Aerial parts	[46]
*rel*-(*1S,2S,3S,4R,6R*)-3-*O*-(6-*O*-caffeoyl-*β*-D-glucopyranosyl)-1,6-epoxy menthane-2,3-diol	Aerial parts	[46]
Siliphioside E	Aerial parts	[46]
(*2E,6E*)- 2,6,10-trimethyl-2,6,11-dodecatriene-1,10-diol-1-*O*-*β*-D-glucopyranoside	Aerial parts	[46]
(*2S*)-1-*O*-Stearoyl-3-O-*β*-D-galactopyranosyl-sn-glycerol	Aerial parts	[34]
(*2S*)-3-*O-*(*9Z,12Z*-Octadecadienoyl) glyceryl-*O*-*β*-D-galactopyranoside	Aerial parts	[34]
Bidensmenthoside A	Whole plant	[50]
Bidensmenthoside B	Whole plant	[50]
11*β*,17-Dihydroxy-beyer-15-ene	Whole plant	[44]
4*β*- Hydroxy-guai-10(14),11(13)-dien-12-oic acid	Whole plant	[44]

## Data Availability

No new datasets analyzed or generated during the study.

## Abbreviations

CCl_4_	Carbon Tetrachloride
DGAT	Diacylglycerol Acyltransferase
DPPH	1,1-Diphenyl-2-Picrylhydrazyl
EAC	Ehrlich Ascites Carcinoma
GPT	Glutamine-Pyruvate Transaminase
GOT	Glutamic-Oxaloacetic Transaminase
HCV	Hepatitis C Virus
HRMCs	Human Renal Mesangial Cells
IC_50_	Inhibition Concentration 50%
MBC	Minimum Bacterial Concentration
MIC	Minimum Inhibitory Concentration
MTT	3-(4,5-Dimethylthiazol-2-yl)-2,5-diphenyltetrazolium bromide
NF-κB	Nuclear Factor κB
RBC	Red Blood Cells
TFC	Total Flavonoid Content
TG	Triglycerides
TPC	Total Phenolic Content
TBARS	Thiobarbituric Acid Reactive Substances
WBC	White Blood Cells
